# A Unique Human Norovirus Lineage with a Distinct HBGA Binding Interface

**DOI:** 10.1371/journal.ppat.1005025

**Published:** 2015-07-06

**Authors:** Wu Liu, Yutao Chen, Xi Jiang, Ming Xia, Yang Yang, Ming Tan, Xuemei Li, Zihe Rao

**Affiliations:** 1 School of Life Sciences, University of Science and Technology of China, Hefei, Anhui, China; 2 National Laboratory of Biomacromolecules, Institute of Biophysics, Chinese Academy of Sciences, Beijing, China; 3 Division of Infectious Diseases, Cincinnati Children's Hospital Medical Center, Cincinnati, Ohio United States of America; 4 University of Cincinnati College of Medicine, Cincinnati, Ohio, United States of America; University of Nantes INSERM, U892, FRANCE

## Abstract

Norovirus (NoV) causes epidemic acute gastroenteritis in humans, whereby histo-blood group antigens (HBGAs) play an important role in host susceptibility. Each of the two major genogroups (GI and GII) of human NoVs recognizes a unique set of HBGAs through a distinct binding interface that is conserved within a genogroup, indicating a distinct evolutionary path for each genogroup. Here, we characterize a Lewis a (Le^a^) antigen binding strain (OIF virus) in the GII.21 genotype that does not share the conserved GII binding interface, revealing a new evolution lineage with a distinct HBGA binding interface. Sequence alignment showed that the major residues contributing to the new HBGA binding interface are conserved among most members of the GII.21, as well as a closely related GII.13 genotype. In addition, we found that glycerol inhibits OIF binding to HBGAs, potentially allowing production of cheap antivirals against human NoVs. Taken together, our results reveal a new evolutionary lineage of NoVs selected by HBGAs, a finding that is important for understanding the diversity and widespread nature of NoVs.

## Author Summary

Human norovirus (huNoV) has diverged into two major lineages (GI and GII) selected by the host histo-blood group antigens (HBGAs). Both lineages further diverge into various sub-lineages (genotypes) that recognize different ABH and Lewis antigens through a common HBGA binding interface shared among strains within each genogroup. In this study, through X-ray crystallography of the P domain of a GII.21 huNoV (OIF) we identified a unique lineage in GII consisting of GII.13 and GII.21 genotypes that recognize HBGAs through a binding interface distinct from the GII conventional binding interface. While the mechanism remains unknown, our finding raises an alert on future emergence of new lineages by the same way via developing new receptor binding interfaces, as well as further divergence of this new lineage into more sub-lineages recognizing different HBGAs, which may impact future epidemiology and strategies for disease control and prevention against huNoVs.

## Introduction

Noroviruses (NoVs) are a group of non-enveloped, single stranded, positive-sense RNA viruses that constitute the *Norovirus* genus in the family *Caliciviridae*. NoVs are genetically diverse, containing six genogroups (GI to GVI) with over 35 genetic genotypes. NoVs exhibit wide host tropisms causing diseases in various mammalians including human. Human NoVs (huNoVs), consisting of mainly GI and GII NoVs, are the most important viral cause of epidemic acute gastroenteritis in humans [1], claiming over 200,000 lives each year [2]. NoVs are encapsulated by a protein capsid that is assembled by a single major structural protein, the capsid protein VP1. Each NoV capsid contains 180 copies of VP1, which are organized as a T = 3 icosahedron [3]. VP1 is divided into the N-terminal shell (S) and the C-terminal protruding (P) domain, forming the interior shell and the multiple protrusions of the capsid, respectively [3]. The P domain can be further divided into P1 and P2 subdomains, corresponding to the head as the outermost portion of NoVs, and the leg of the protrusion, respectively. Compared with S and the P1 domain, the P2 domain exhibits the most variable sequences, which are responsible for strain-specific virus-host interactions and immune responses of NoVs.

HuNoVs interact with histo-blood group antigens (HBGAs) in a strain-specific manner [4, 5]. HBGAs are fucose-containing glycans in specific sequences as determinants of human and animal blood types, including A/B/O, secretor (H), and Lewis (Le) or non-secretor (H negative) types. They are often present as parts of the carbohydrate moiety of cell surface glycoproteins and glycolipids with N- or O-linkage [6]. The biological roles of HBGAs in huNoV infection have been revealed by human volunteer challenge studies [7–9] and outbreak investigations [10, 11] of huNoVs, in which an association between the host susceptibility and the HBGA binding patterns of huNoVs has been established.

Despite the recent breakthroughs in culturing huNoVs in BJAB cells [12], the use of huNoV reverse genetics system [13], and the development of an immunocompromised mouse model [14] for huNoV propagation, an effective cell culture system or an animal model for huNoVs remains lacking. As a result, our understanding on huNoV-HBGA interactions relies mainly on data from *in vitro* studies using various recombinant subviral particles as models of huNoVs. Virus-like particles (VLPs), which are produced by expression full-length VP1 via a eukaryotic expression system, share similar structures and functions with the capsid of a native virion, and they have been used extensively as a huNoV surrogate. In addition, smaller P domain complexes that are self-assembled by expression of the huNoV P domain, including the P dimer [15], small P particle [16] and P particles [17, 18], are also used for the study of huNoV-HBGA interactions. Knowledge on the structures of the HBGA binding interfaces has been derived mainly from crystal structures of P domain dimers in complex with HBGA oligosaccharides [19–26], by taking advantage of its small size (~69 kDa) and easy production.

**Fig 1:**
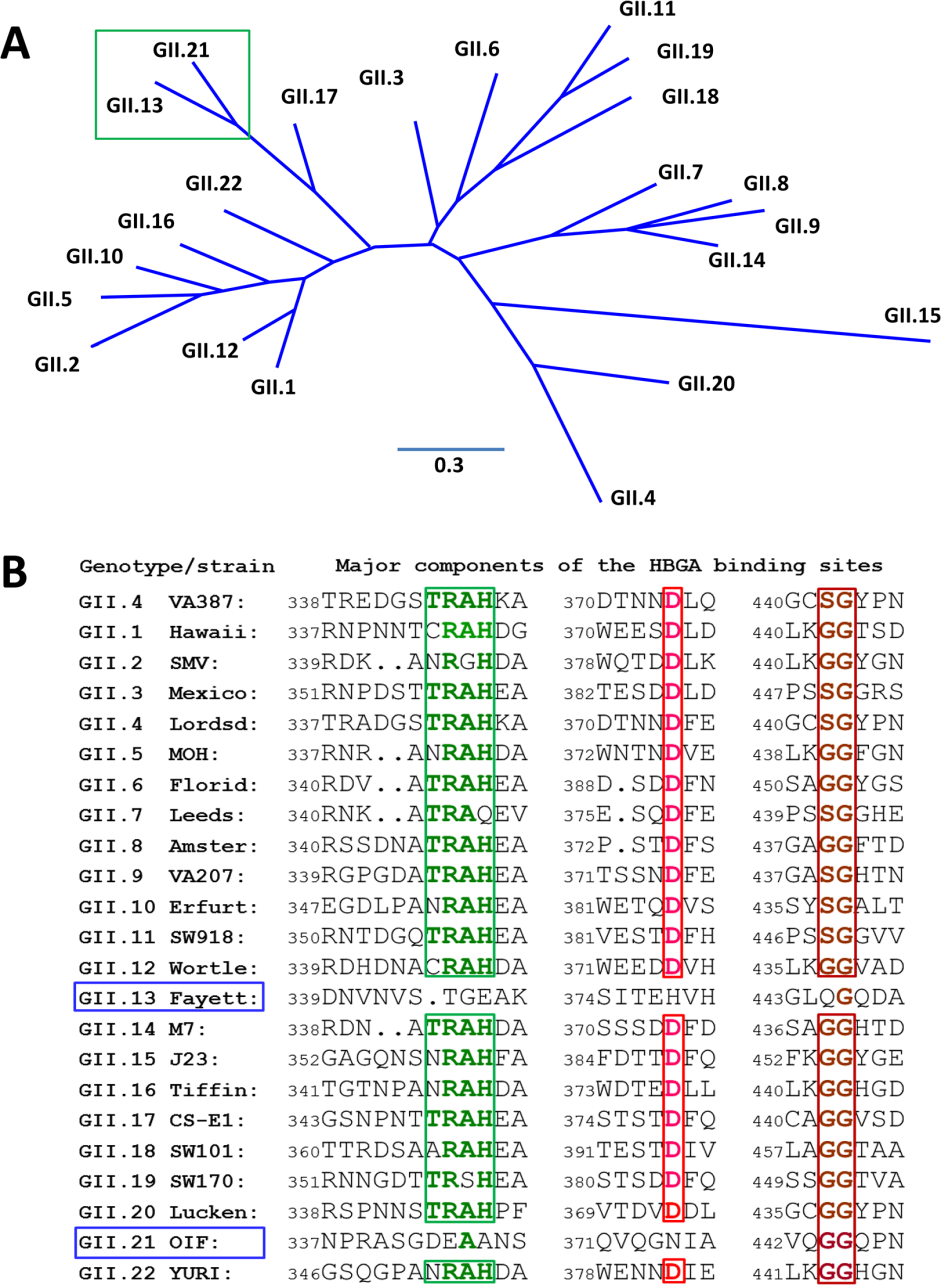
Phylogenetic and sequence analysis of genogroup (G) II noroviruses (NoVs). (A), Phylogenetic tree of GII NoVs that is re-drawn according to [52]. The unique genetic branch consisting of GII.13 and GII.21 genotypes is indicated by a green frame. (B), P domain sequence alignment of NoVs representing each of the 20 GII genotypes with a focus on the residues at the HBGA binding interface. The conserved amino acids constituting the GII conventional HBGA binding interface are indicated with colored letter in frames. The GenBank accession numbers of the P domain sequences are: AY038600.3 (VA387), U07611 (Hawaii), AY134748 (SMV), U22498 (Mexico), X86557 (Lordsdale), AF397156 (MOH), AF414407 (Florida269), AJ277608 (Leeds), AF195848 (Amsterdam), AAK84676 (VA207), AF427118 (Erfurt), AB074893 (SW918), AJ277618 (Wortley), AY113106 (Fayettevil), AY130761 (M7), AY130762 (J23), AY502010 (Tiffin), AY502009 (CS-E1), AY823304 (SW101), AY823306 (SW170), EU373815 (Lucken), AY675554 (OIF), and AB083780.1 (YURI).

Structural analysis of known HBGA binding interfaces of huNoVs showed that GI and GII huNoVs recognize HBGAs through a conserved, genogroup-specific binding interface (reviewed in [27–29]), suggesting a strong selection of huNoV evolution by human HBGAs. On the other hand, the GI and GII HBGA-binding interfaces are distinct in the locations, structures, residue compositions, and HBGA binding modes [27–29], indicating a long separation of the two genetic lineages. In this study we report a new evolutionary lineage consisting of GII.13 and GII.21 genotypes within GII, which does not share the conventional GII HBGA binding interface (Fig 1), but remains binding ability to HBGAs [5, 30]. X-ray crystallography of the GII.21 OIF virus P domain complexed with a Le^a^ antigen revealed a new HBGA binding interface that is distinct from the GII conventional binding interface. Sequence alignment further showed that the amino acid composition of the new binding interface of OIF is highly conserved among all members of both GII.21 and a closely related GII.13 genotype. These results indicate that the genetic branch consisting of GII.21 and GII.13 developed along a novel evolutionary path that split from the mainstream lineage of GII NoVs selected by HBGAs. While many questions on the cause and mechanisms behind the emergence of this new lineage remain unanswered, our data point towards a continual occurrence of new lineages, which may significantly impact future epidemiology and prevention strategies against huNoVs.

## Results

### Crystal structure of the native OIF P domain protein

**Fig 2:**
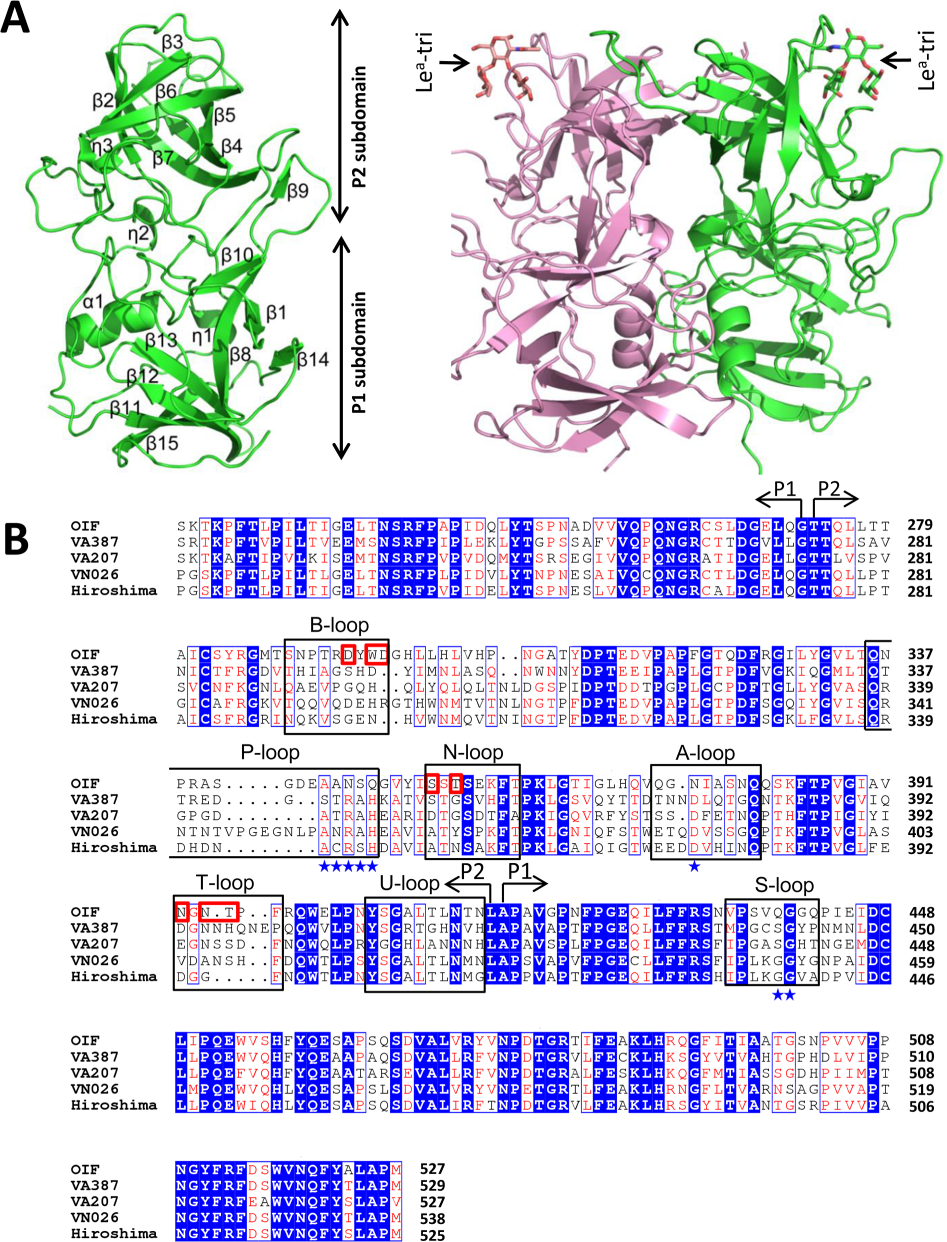
Structures (A) and the structure-based sequence alignment (B) of the P domains of OIF virus. (A), Overall structure (ribbon representation, green) of OIF P protein monomer (left) and its dimeric form (ribbon representation, green and pink) in complex with Le^a^ trisaccharide (stick representation, indicated by arrows) (right). Secondary structural elements are labeled in the P monomer (left). (B), the structure-based sequence alignment of the P domains of GII.21 OIF, GII.4 VA387, GII.9 VA207, GII.10 VN026 and GII.12 Hiro. Regions spanning the P1 and P2 subdomains are indicated by arrows. Identical residues are highlighted with blue background, while similar residues are shown in red characters. Residues forming the HBGA binding interface of the GII.21 OIF are indicated in red frames, while the conserved amino acid residues forming the conventional GII HBGA binding interface are indicated by blue stars. The seven surface loops that constitute the two types of HBGA binding interfaces are indicated by black frames. The letters in blue backgrounds indicate the identical amino acid sequences, while the red letters indicate the similar amino acids among the five NoVs. Blue box fames both identical and similar residues.

The crystal of the native P domain protein belongs to the P2_1_ space group, with two monomers forming a dimer in an asymmetric unit. The final refined structure of native P domain includes residues 223 to 527, with the exception of a loop region comprising residues 340~342 due to the lack of recognizable electron density. While the P domain of OIF virus shares only ~50% sequence homology with other GII P domains of known crystal structures, it shows an arrangement of overall and secondary structures similar to those huNoV P proteins, including GII.4 VA387 [20], GII.10 Vietnam 026 [23], GII.9 VA207 [21], and GII.12 Hiro [23] with Cα atoms r.m.s.d. = 0.85 Å, 0.83 Å, 0.82 Å, and 0.62 Å, respectively. Like other huNoVs, the OIF P domain has two moieties (Fig 2), with the inner portion or P1 subdomain (residues 223 to 272, and 416 to 527) constituting the leg, and the outer moiety or P2 subdomain (residues 273 to 415) forming the protruding head of the P dimer.

**Fig 3:**
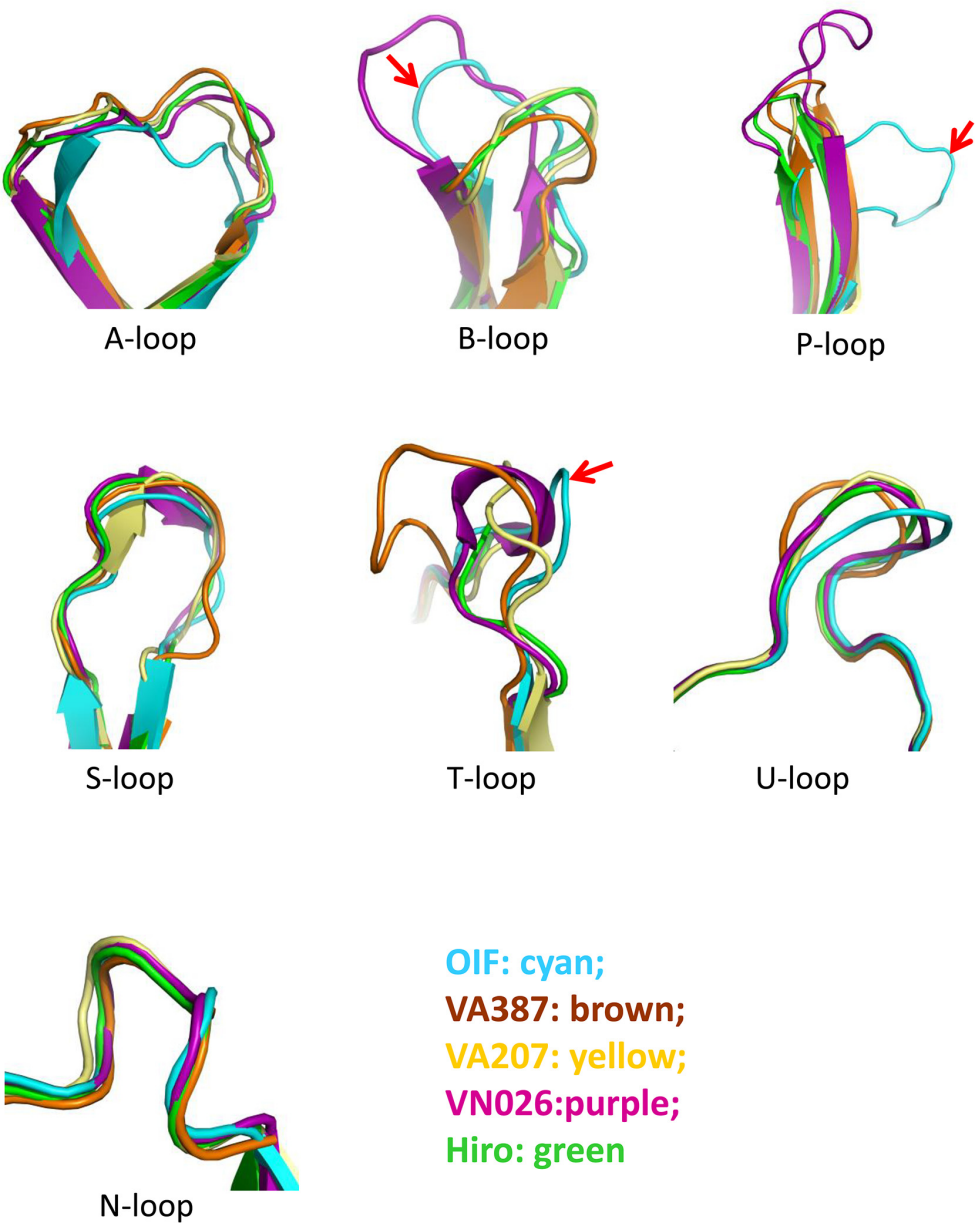
Comparison of the seven major surface loops of OIF virus with those of five other GII noroviruses. Color schemes: cyan, OIF virus (GII.21); brown, VA387 (GII.4); yellow, VA207 (GII.9); purple, Vietnam026 (VN026, GII.10); green, Hiro (GII.12).

**Fig 4:**
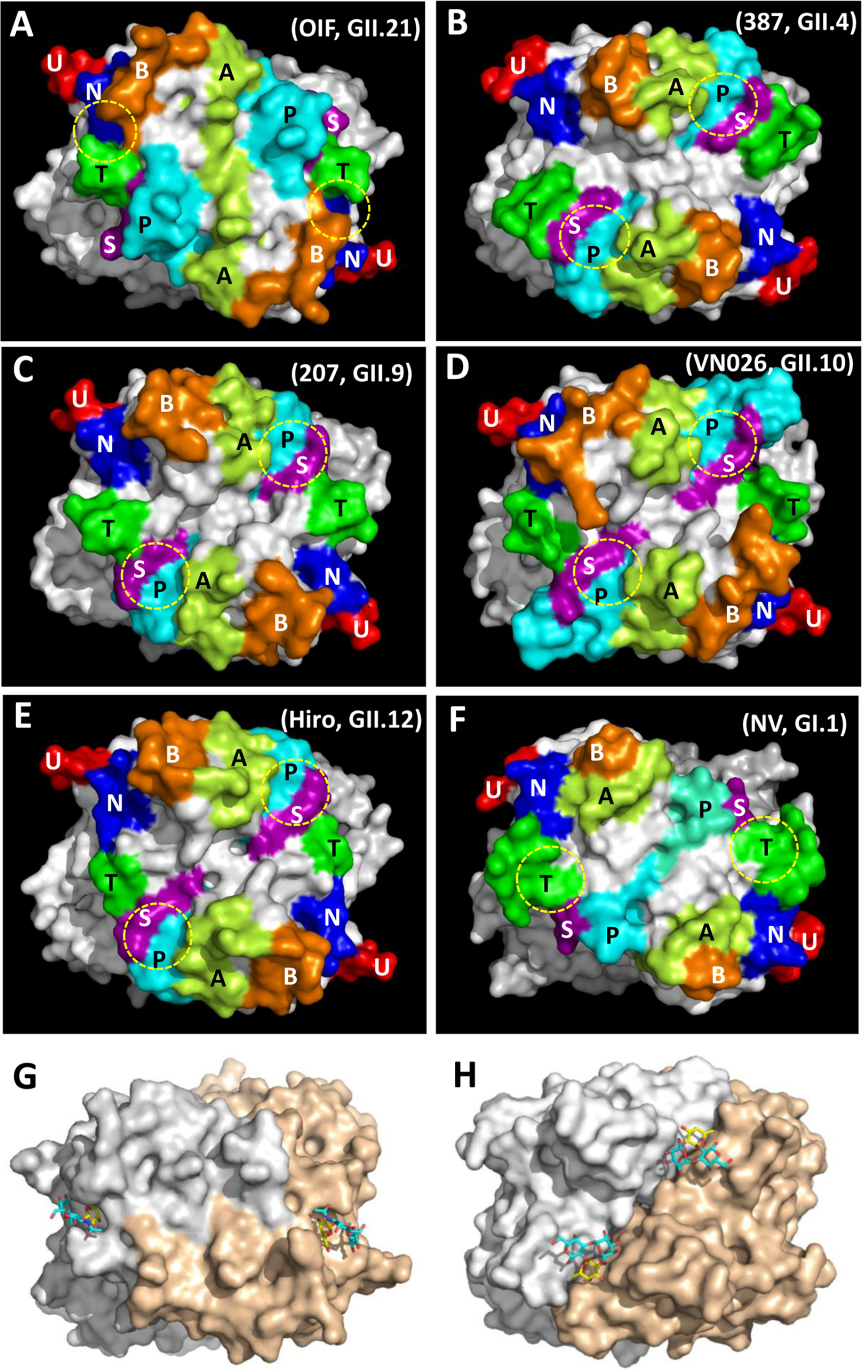
Evolutionary changes of the topologies of the P dimer top surface of OIF compared with other noroviruses. (A), GII.21 OIF virus; (B), GII.4 VA387 (387); (C), GII.9 VA207 (207); (D), GII.10 Vietnam026 (VN026); (E), GII.12 Hiro; (F), GI.1 Norwalk virus (NV). The seven major surface loops, i.e. A-, B-, P-, S-, T-, U-, and N-loops are indicated in light-green, orange, cyan, purple, dark-green, red, and blue, respectively. The locations of the HBGA binding interfaces are indicated by yellow dashed circles. (G and H), Comparison of the top surface structures of the P dimers of OIF virus (G) and VA387 (H). The two P protomers are labeled in grey and sand colors. Bound HBGAs are shown in stick representation, with the major binding saccharide (galactose in OIF, fucose in VA387) colored in yellow.

The two monomers of the OIF P dimer are related by a non-crystallographic two-fold axis, which forms the biologically active protrusion of the NoV capsid. The P dimer has a dimension of 55 Å×64 Å×70 Å with an extensively buried interface of 3,500 Å^2^ between two protomers, including hydrophobic and hydrophilic interacting residues from both P1 and P2 subdomains (Fig 2A). These extensive inter-molecular interactions contribute to the stability of the P dimer. Although the OIF P dimer shares similar global structures with the previously reported P dimers of other huNoVs, significant differences on the top surface are clearly seen, mainly due to the differences in the sequences, lengths, and conformations of several surface loops (Figs 3 and 4) (see below).

### Top surface structure of the P dimer

The P-loop of the OIF P dimer exhibits a unique conformation, taking a ~90° flip (Fig 3), which results in its extruding out of the flat surface and approaching the opposite protomer. This leads to greater exposure of the P-loop on the top surface of the P dimer compared to that in other GII huNoVs (Fig 4). As a result, the majority of the S-loop is covered in the OIF virus. Such structural rearrangements destroy the structural integrity of the conventional GII HBGA binding interfaces that are formed mainly by P- and S-loops (Figs 2 and 4). On the other hand, the B- and the T-loops also exhibit unique conformations (Fig 3), approaching to each other in closer proximity with the N-loop (Fig 4A). This special structural feature allows the formation of new HBGA binding interface (see below), formed by the B-, T- and N-loops, a scenario that is absent in all other known GII huNoV structures (Fig 4). The protrusions of the P- and A-loops directed towards the opposite protomer restructure the boundary between two P monomers into a saw tooth-like structure, in contrast to the straight line-like pattern in other GII NoVs (Fig 4, compare G and H). These different surface structures and conformations of OIF P dimer might also contribute to the difference in antigenic and immunological properties of GII.21 compared to other huNoV genotypes.

### The HBGA binding interface

**Fig 5:**
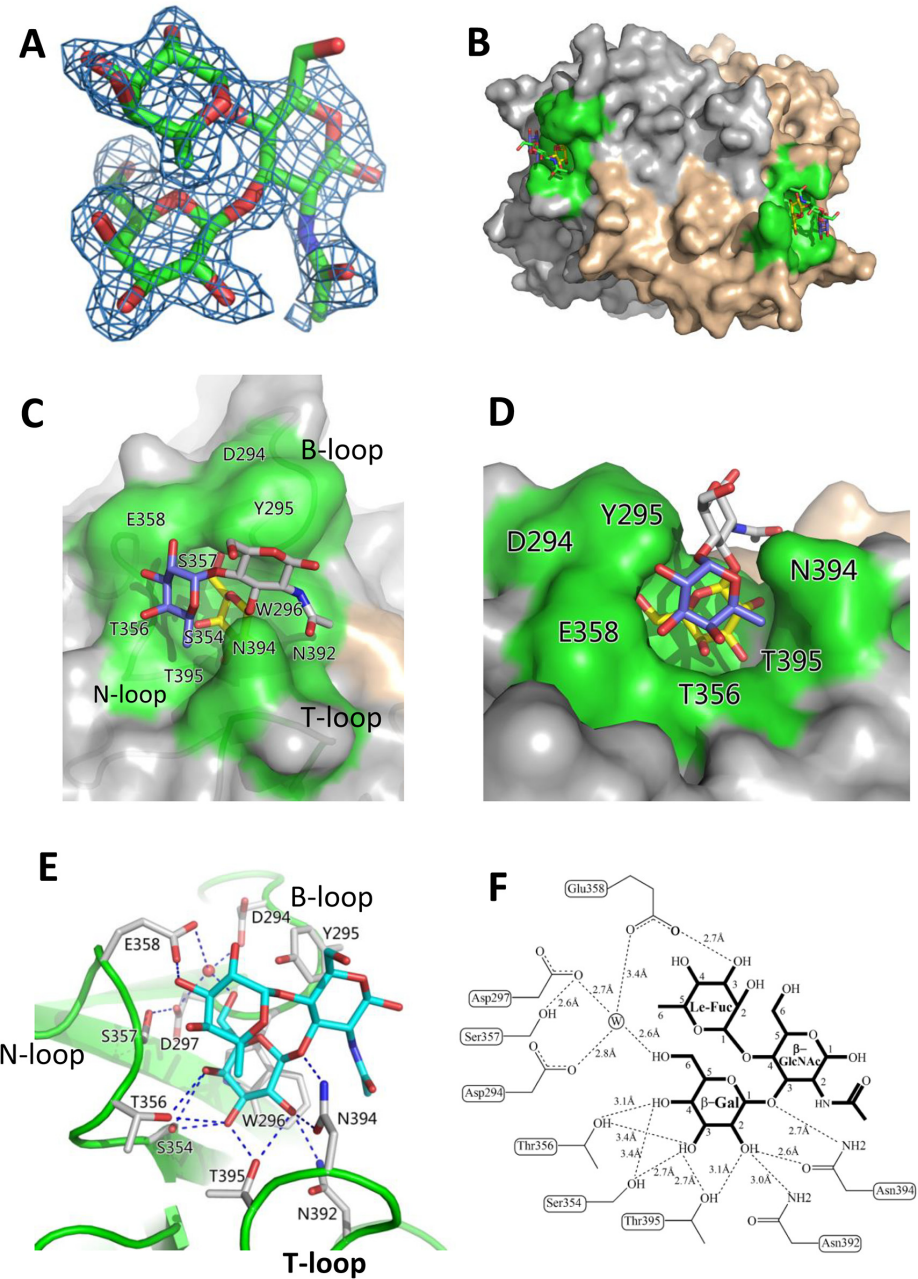
Identification of the HBGA-binding interface of OIF virus and its interaction with the Le^a^ trisaccharide. (A), (Fo-Fc) omit electron density map of Le^a^ trisaccharide. The omit map was created using the final structure of OIF P domain without Le^a^ trisaccharide, and the mesh map was contoured at 2.0 sigma (blue) around the selection site, with a coverage of 1.6Å radius. Carbon, oxygen and nitrogen atoms are indicated in green, red and blue, respectively. (B), Surface representation of the P dimer of OIF virus in complex with Le^a^ trisaccharide (stick representation). (C and D), Close-ups of top (C) and side (D) views of the HBGA binding interface (surface representations) with indications of the residues that form the HBGA binding interface (colored in green). The Le^a^ trisaccharide (stick representation) is also indicated. The individual saccharides of the Le^a^-trisaccharide are colored differently: β-Galactose, yellow; α-Fucose, purple; and N-acetyl glucosamine, grey. (E and F), the interacting networks between the side/main chains of the amino acids at the binding interface with the β-Galactose (Gal) and α-Fucose (Fuc) (Le-Fuc). The hydrogen bonds are indicated by dashed lines. The water molecule is presented as a red ball (E) or a circled W (F).

As shown in previous studies [5, 30], the OIF virus that does not share the conventional GII HBGA binding interface only recognizes Le^a^ antigen. To understand their binding mechanism, we co-crystallized the OIF P domain with Le^a^ trisaccharide (Le^a^-tri). The P domain-Le^a^-tri complex was crystallized in P2_1_2_1_2_1_ space group, with one homodimer in each asymmetric unit. The Le^a^-tri is clearly visible in the 2Fo-Fc difference electron density map, with all three rings of the Le^a^-tri well fitted into the map (Fig 5A). Two symmetric Le^a^ binding interfaces are identified on the top surface of the P dimer (Fig 5B), each of which is formed by eleven residues from the P2 domain of a single protomer. Specifically, residues W296 from the B-loop, S354 and S357 from the N-loop, N392 and T395 from the T-loop form the depressed region at the bottom of the binding pocket (Fig 5C–5E). We noticed that, although S357 does not interact directly with the Le^a^-tri, it forms a strong hydrogen bond (2.6Å) with the D297 to stabilize the side chain of the latter, ensuring the structural integrity of the binding pocket (Fig 5E and 5F). The surrounding wall of the binding pocket is built by D294 and Y295 from B-loop, T356 and E358 from N-loop, and N394 from T-loop (Fig 5C–5E). None of these residue compositions are conserved with those of the known GII HBGA binding interfaces that is contributed by residues from P-, S-, and A-loops (Figs 2 and 4). The OIF HBGA binding interface also differs completely from those of the GI huNoVs (Fig 4F). Thus, this OIF binding interface represents a previously unrecognized HBGA binding interface.

### The β-Gal binding site

The HBGA binding pocket interacts with Le^a^ antigen through the β-galactose (β-Gal), one of the two saccharides of the precursor disaccharide, as the major binding saccharide (MaBS). The β-Gal is firmly held inside the binding pocket by nine direct hydrogen bonds formed by the side chains of S354, T356, N392, N394 and T395, in which the side chains of S354 from the bottom and N394 from the wall of the pocket form three strong hydrogen bonds (2.7 Å or 2.6 Å) with β-Gal (Fig 5E and 5F). It is noteworthy that the side chain of W296 is oriented nearly in parallel with the β-Gal ring, which leads to a hydrophobic interaction/stacking with the latter to further support the interaction between β-Gal and the binding pocket. In addition, the side chains of D294, D297 and E358 form water-bridged hydrogen bonds with β-Gal via a common water molecule, contributing to the binding outcomes between the OIF P domain and the Le^a^ antigen. Together, these amino acids form the β-Gal binding site that is the major component of the HBGA binding interface of OIF virus.

### Le Fuc binding site

As a result of the strong interactions between the β-Gal and the binding pocket, the Lewis fucose (Le-Fuc, α-1,4-fucose) is orientated between the walls of N394 and E358. Only a single direct hydrogen bond (2.7 Å) forms between E358 and the Le-Fuc (Fig 5E and 5F) and therefore, the Le-Fuc is a minor binding saccharide (MiBS). As a result of the β-Gal/Le-Fuc-binding pocket interactions, the N-acetyl-β-glucosamine (β-GlcNAc), the other saccharide of the precursor disaccharide, is pointing away from the surface of the binding interface. No hydrogen bond was observed between β-GlcNAc and the binding interface (Fig 5C–5F).

### Validation of the HBGA binding interface

**Fig 6:**
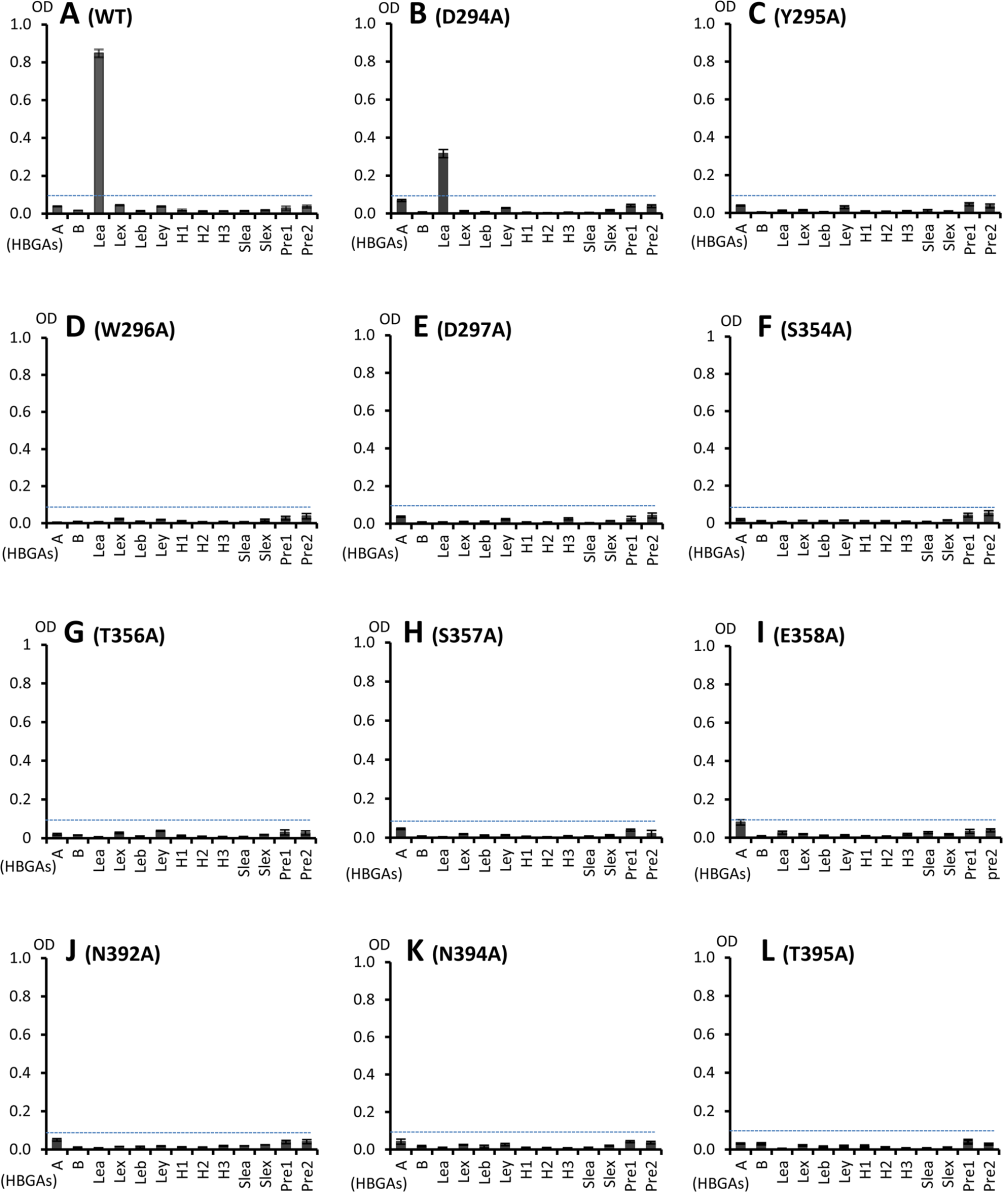
HBGA-binding of wild type and various mutant P particles with single amino acid mutations at the HBGA-binding interface of OIF virus. (A), Binding of wild type OIF P particles with a panel of oligosaccharides representing different HBGAs (A, B, Le^a^, Le^b^, Le^x^, Le^y^, H1, H2, H3, sialyl Le^a^ and sialyl Le^x^) and the type 1 (Pre1) and type 2 (Pre 2) precursors. (B to L), Binding of various mutant P particles with single amino acid mutations at the HBGA-binding interface with the same panel of oligosaccharides as in (A). The concentrations of the P particles were 10 μg/ml, while oligosaccharides for plate coating at 2 μg/ml. Y axes indicate the binding signals in optical densities at 450 nm (OD450), while the X-axes indicate different oligosaccharides.

We further investigated the roles of the eleven residues that form the HBGA binding interface of the OIF virus using single-point mutagenesis (Figs 5 and 6). Compared with the wild type P particle of OIF virus that binds strongly to Le^a^ oligosaccharide, but not other oligosaccharides representing different HBGA types, including precursors (Fig 6A), mutant P particles with a single mutation at one of those amino acids to an alanine lost the binding to Le^a^ antigen completely (Fig 6C–6L) or nearly completely (Fig 6B). These results confirmed the importance of these residues for the structural and functional integrity of the HBGA binding interface. In addition, except for a slight increase in binding to the A antigen of the E358A mutant, no changes in binding to other HBGA types were observed for these interface mutants.

### Glycerol binds the HBGA binding pocket of the OIF P domain

**Fig 7:**
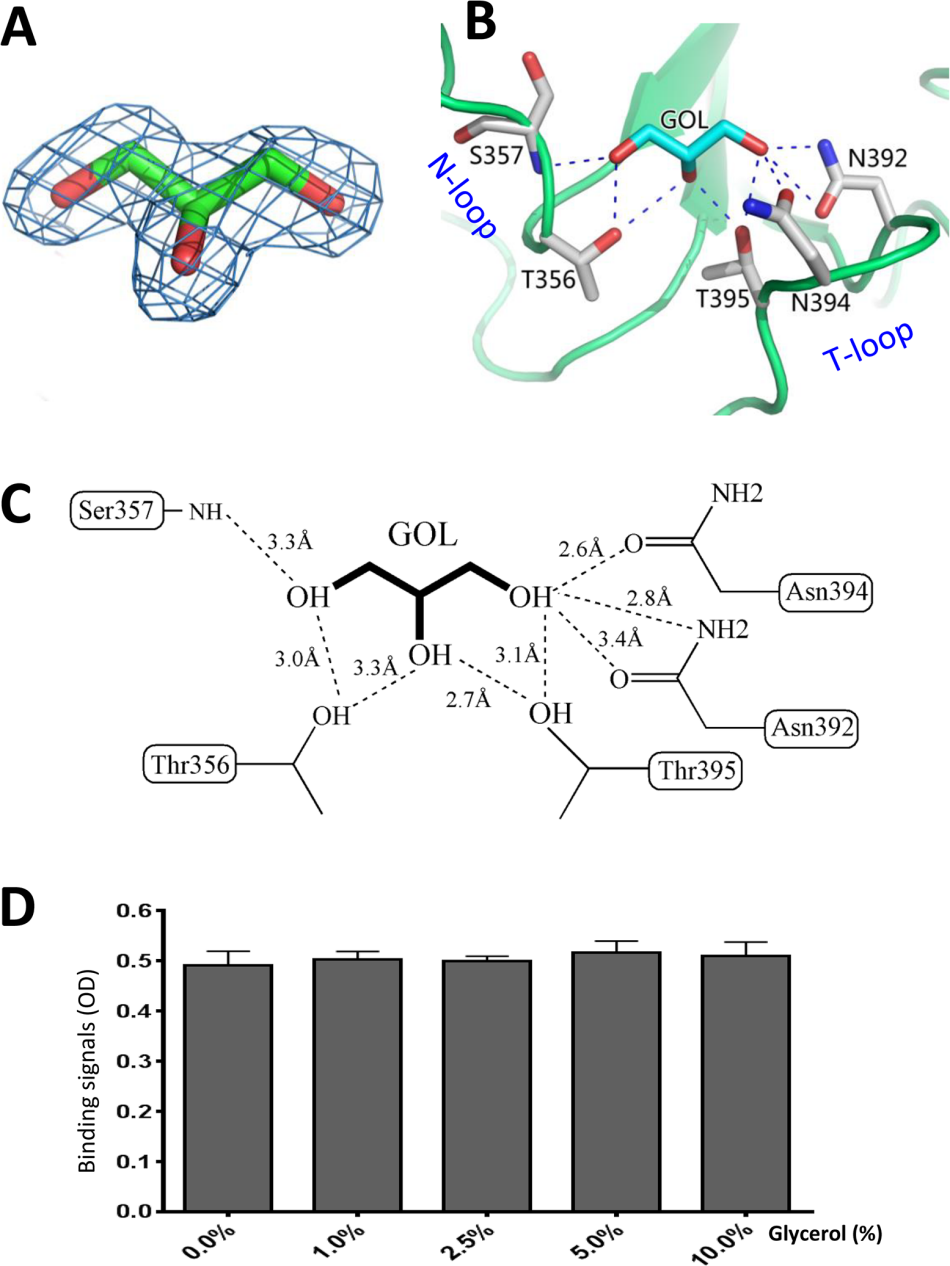
Glycerol binds the HBGA binding interface of OIF virus. (A), (Fo-Fc) omit electron density map of glycerol. The omit map was created and contoured as described in Fig 5A. (B and C), The interacting networks between the side chains of the amino acids of the HBGA binding interface of OIF virus with the glycerol molecule. The hydrogen bonds are indicated by dashed lines. Carbon, oxygen and nitrogen atoms are in cyan, red and blue, respectively. (D) Blocking effects of monomeric glycerol (0–10%, X-axis) against the binding (Y-axis) of the polyvalent Le^a^-tri-PAA (polyacrylamide) conjugates (2 μg/mL) to OIF P particles (4 μg/mL) via ELISA-based blocking assays.

Analysis of the native OIF P domain structure revealed that a glycerol molecule occupies the HBGA binding pocket, which was clearly visible from the 2Fo-Fc omit map (Fig 7A). Originally, glycerol was part of the protease solution acting as a stabilizer during the cleavage of the GST-P domain fusion protein. The glycerol molecule is held firmly inside the HBGA binding pocket by eight direct hydrogen bonds between the hydroxyl groups of the glycerol and the side chains of N392, N394 and T395 from the T-loop, T356 and the main chain of S357 from the N-loop (Fig 7B and 7C). Interestingly, the glycerol molecule resembles partial structures (C2, C3, and C4 with their hydroxyl groups) of the β-Gal that contributes the vast majority of the interactions with the binding pocket (Compare Fig 5F with Fig 7C), thus explaining the observed interactions.

### Glycerol blocks the interaction of OIF P domain with the Le^a^ antigen

We found that inclusion of glycerol in any process of purification or crystallization would prevent the binding of the Le^a^ antigen to the same binding pocket. The later success in identification of HBGA binding interface (Fig 5) of OIF virus using the P protein without glycerol proved our assumption that glycerol occupies the HBGA binding pocket and blocks further interaction of the binding pocket with the Le^a^ antigen. Co-crystallization followed by structural determination using the P protein in the presence or absence of glycerol confirmed our observation. We then performed ELISA binding assays to test if the monomeric glycerol (1–10%) can block binding of polyvalent Le^a^-tri-PAA (polyacrylamide) conjugates (2 μg/mL) to OIF P particles (4 μg/mL). We did not observe any detectable blocking effects (Fig 7D), indicating that the free glycerol can block binding of free, monomeric Le^a^-tri, but cannot inhibit the binding of polyvalent Le^a^-tri molecules to the HBGA binding pocket.

### Conservation of the HBGA binding pocket of the GII.13/GII.21 lineage

**Fig 8:**
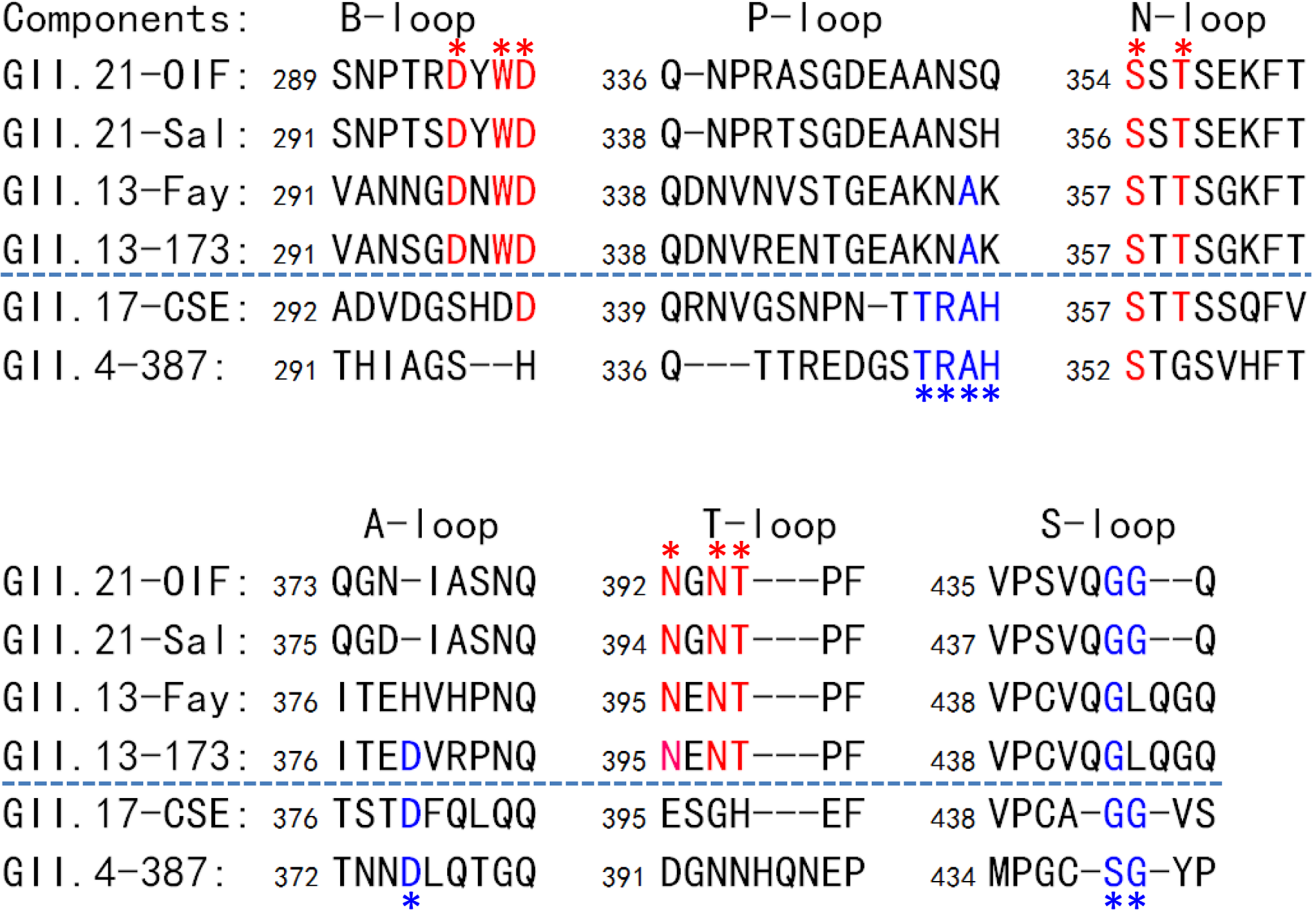
Sequence alignment of the surface loops of the P domains forming the HBGA-binding interfaces. Sequences of the six surface loops forming on the HBGA-binding interfaces representing the two genotypes of the GII.21 and GII.13, as well as GII.17, the genotype genetically close-relating to the GII.13/21 lineage, and GII.4 as a representative of the GII noroviruses (NoVs) with the conventional GII HBGA binding interfaces are aligned. The six surface loops that contribute to the formation of the two types of GII HBGA binding interfaces are labeled. The conserved residues forming the binding interface of OIF virus are shown in red fonts and labeled by red asterisks, while those forming the conventional GII binding interface are shown in blue fonts and labeled by blue asterisks. The GenBank accession numbers of P domain sequences are: AY675554 (OIF), AFC89665.1 (Sal), AY113106 (Fay), JN899242 (173), AY502009 (CSE), AY038600 (387).

The unique HBGA binding interface of the GII.21 OIF virus prompted us to examine whether this is a common feature of this specific huNoV lineage. Representative P domain sequences of GII.13/21 huNoVs were aligned and compared with those of GII.17, which is genetically closest to the GII.13/21 lineage (Fig 1A), as well as with that of GII.4, which is the most prevalent genotype. We focused on the surface loops that form the OIF HBGA binding pocket (B-, T, and N-loops) and the conventional GII binding interface (P-, S- and A-loops) (Fig 8). We found that most members of the GII.13/21 lineage share the major residues that form the novel HBGA binding pocket, indicating a new evolutionary selection has occurred among members of this genetic lineage. In contrast, GII.17 retained the conventional GII HBGA binding interfaces, indicating that the occurrence of the OIF-like binding pocket in the GII.13/21 lineage was after the evolutionary divergence with the GII.17 genotype.

## Discussion

GI and GII NoVs constitute the vast majority of huNoVs, and they were shown previously to interact with HBGAs through two distinct, genogroup-specific HBGA binding interfaces [31]. The two HBGA binding interfaces differ in their locations on the top of the protruding dimer, their residue composition as well as their interacting modes with HBGAs, but are highly conserved among genotypes within each of the two genogroups (reviewed in [27–29, 31]). It is therefore suggested that GI and GII NoVs must split from their common ancestor a long time ago during evolution into two independent evolutionary lineages, from which the two genogroup specific HBGA binding interfaces evolved individually. It is noteworthy that both binding interfaces recognize most, if not all, of the same repertoire of the polymorphic human HBGAs despite their distinct features, indicating that HBGA is an important selection factor in the evolution of huNoVs.

The presence of the two different HBGA binding interfaces in huNoVs raises the question as to whether such an event may occur again during evolution, especially within the same genogroup. We noted earlier on that a genetic branch that consists of GII.13 and GII.21 genotypes does not share the residue composition of the HBGA binding interface of the mainstream GII NoVs, but maintains HBGA binding function [5, 30], suggesting that this GII.13/21 lineage may interact with HBGAs through an unknown binding interface. By performing X-ray crystallography of the native P domain of the GII.21 OIF virus and its complex with Le^a^ trisaccharide (Le^a^-tri), we found that GII.21 huNoVs recognize HBGAs via a previously unrecognized binding interface. The newly identified HBGA binding interface is spatially, structurally, and compositionally distinct from the well-studied conventional GII and GI HBGA binding interface.

The conventional GII HBGA binding interface is formed by three surface loops, i.e. the P- and A-loops from the P2 subdomain and the S-loop from the P1 subdomain. The binding interface is located directly on the boundary between the two P protomers, which makes each binding interface bivalent contributed by residues from both P protomers. In the GII.21 OIF virus, however, the conventional HBGA binding interface has been clearly abolished by the rearrangement of the three loops. Through a 90 degree flip, the P-loop extends to the opposite protomer, covering the vast majority of the S-loop and moving away from the A-loop. Nevertheless, this dramatic structural rearrangement of the OIF P dimer also helps to reconstruct a new HBGA binding interface, formed by other three surface loops, i.e. the B-, N- and T loops from the P2 subdomain of a single P protomer. We also noted that the binding interface corresponding to that of OIF virus does not exist in the mainstream GII NoVs, because the B- and T-loops are far away from each other in those GII NoVs.

In addition to the location, residue composition, and structure, the two GII HBGA binding interfaces are also different in their binding modes to HBGAs. The general binding modes of huNoVs have recently been summarized [28, 29]. GI NoVs bind HBGAs via a Gal (theα- or the β-Gal), while GII NoVs bind via a Fuc (the α-1, 2/3/4 Fuc) as the major binding saccharide (MaBS). The OIF virus interacts with the Le^a^ antigen via the β-Gal as the MaBS, distinct from the convention GII, but similar to the GI binding interfaces. In addition, the OIF-Le^a^ interaction relies mainly on the β-Gal that contributes 11 out of total 12 hydrogen bonds, including 9 direct ones. The α-Fuc (Le-Fuc) contributes only one hydrogen bond, while the β-GlcNAc does not participate directly in binding. This “upright” binding mode is also different from the “flat” mode observed in the conventional GII binding interfaces, which often includes two saccharides as the minor binding saccharides (MiBS), which usually contribute three or more interacting bonds. Again, the binding mode of OIF virus to HBGA appears to be more similar to that of GI NoV than to GII NoV [28, 29].

Our crystal structures of the HBGA binding interface of OIF virus complexed with Le^a^-tri reveal why OIF virus does not bind H, A, and B antigens. The H epitope (α-1,2 Fuc) and the A/B epitope (α-GalNAc/Gal) are linked to the 2’ and 3’ hydroxyl groups of β-Gal, respectively [29]. However, both the 2’ and 3’ hydroxyl groups of the β-Gal are located at the bottom of the binding pocket (Fig 5). Any addition of an extra saccharide at one of these positions would cause serious steric hindrance, and thus prevent the binding of H, A, and B antigens to the binding pocket of the OIF virus. This binding mode may also explain the absence of changes of the binding specificity through the eleven single residue mutations at the amino acids forming the OIF binding interfaces (Fig 6). This HBGA binding interface and its binding mode appear not to provide much flexibility to different HBGAs. Thus, this scenario of OIF virus differs clearly from observed flexibility of the conventional GII binding interface that is capable to bind more than one HBGA type and that can change binding specificity through a single residue mutation in or around the binding interface [32–36].

The formation of the new HBGA binding interface in the GII 13/21 lineage also raises questions as to how the conventional GII HBGA binding interface has been abandoned, and how a new interface was formed. The huge structural variation of the conventional GII binding interface in OIF virus may represent the accumulation of multiple gradual changes over time, following the acquisition of the novel binding interface and loss of binding function of the old one and HBGA selection. The movements of the B-, T- and N-loops to form the new HBGA binding interface can be monitored through the huNoVs with known crystal structures (Fig 4). The three loops are far away in GI.1 (Norwalk virus), GII.4 (VA387), GII.9 (VA207), GII.12 (Hiro), but are closer sterically in GII.10 (Vietnam026). Thus, movements of these loops are the structural basis for the formation of the functional HBGA binding interface found in OIF virus, although how such a functional binding interface was created in detail remains to be elucidated.

How the GII.13/21 lineage with the new HBGA binding interface split from the GII.17 genotype with the conventional one remains unclear. The capsid proteins (VP1s) of the GII.13/21 lineage share generally ~75% sequence identify with that of the GII.17 NoVs and these differences concentrate in their P domains that shows only ~62% sequence identity. This great sequence difference indicates that the two lineages have split from each other for a long time, which makes an attempt to reconstruct their common ancestor difficult. We speculate that the GII.17 P domain must have similar surface topology and loop organization to those of the mainstream GII NoVs due to the fact that the GII.17 NoV retains the conventional binding interface. In contrast, the GII.21 OIF shows very different surface topology and loop arrangements compared with the other GII NoVs, leading to the formation of the new HBGA binding interface. Unfortunately, the lack of the crystal structures of a GII.17 P domain prevents our further understanding on the evolutionary scenarios of the separation between the GII.13/GII.21 lineage and the GII.17 genotype. Thus, future study to solve the structures of a GII.17 P domain is of significance.

While the conservation of the conventional GI and GII HBGA binding interfaces indicates that there was selection pressure imposed by HBGAs functioning as attachment factors or receptors, the conservation of the HBGA binding interface of the GII.13/21 lineage (Fig 8) also indicates that new selection pressure of HBGA for the new binding interface has been established. Thus, it is plausible to expect that the conserved binding interface of the GII.13/21 lineage will continue to be maintained, just like the GI and GII binding interfaces. It would be important to show whether different strains of the GII.13/21 lineage can also recognize different HBGAs, just as GI and GII NoVs do. It was noted that Y295 in the binding pocket of GII.21 OIF is replaced by an asparagine (N) in GII.13 (Fig 8) that is highly conserved among most known GII.13 isolates [37]. It would be of significance to assess how this mutation could affect the HBGA binding profiles of GII.13 NoVs. In addition, N392 is another important residue for the structural and functional integrity of the GII.21 OIF binding interface, which was confirmed by the loss of the binding function through a N392A mutation (Fig 6J). This residue is also conserved among GII.13 NoVs (N395, Fig 8). However, we noted an exception, a GII.13 isolate called Kashiwa 47 (BAC05515) has an N395D mutation [37] and the recombinant VLPs of this isolate did not bind human saliva samples and tested synthetic HBGAs [38], consistent with our result of N392A mutation in GII.21 OIF.

Members of the GII.13/21 lineage revealed increased prevalence in the recent years. Surveillance indicates that the GII.13 and GII.21 huNoVs continue to cause outbreaks in the USA (CaliciNet http://www.cdc.gov/norovirus/reporting/calicinet/data.html), Europe (NoroNet, http://www.rivm.nl/dsresource?objectid=rivmp:248062&type=org&disposition=inline), and other countries [37, 39, 40], indicating that the GII.13/21 lineage is of clinical importance. It was also noted that, after being silent for the past decades, GII.17 NoVs became dominant in southern China in the past winter [41], overwhelmed GII.4 NoVs. Thus, continual surveillance of the epidemiological trends of the GII.13/17/21 lineage will be necessary in the future.

The fact that a new functional carbohydrate binding interface can be created through evolution raises the possibility of zoonotic transmission of NoVs. A new binding interface could recognize different host factors or receptors among human populations or animal species. The currently known host tropisms of different human and animal NoVs may be examples of such possibility. The genetic branch of porcine NoVs, consisting of the GII.11, GII.18 and GII.19 genotypes may be another example. Since these porcine NoVs share the conventional GII binding interface, even though they do not infect humans, it would be important to determine what host factor that porcine NoVs may recognize and understand how such molecule(s) interact with the conserved GII binding interface of porcine NoVs.

Another important finding of this study is the strong interaction of the HBGA binding pocket with a glycerol molecule, a fact mirrored by its inhibition of OIF P domain interaction with Le^a^ antigen. Thus, glycerol, a small, low cost compound, may be a promising antiviral candidate against infection of the GII.13/21 NoVs. The glycerol molecule shares the structure of the β-Gal that is involved in the interaction to the binding pocket. A similar scenario was also observed in a previous study showing that a citrate molecule occupies the binding pocket of a GII.10 NoV (Vietnam026) [42] and importantly, does so with similar binding affinity observed between HBGAs and GII.10 P domains. However, our results showed that the free, monomeric glycerol cannot inhibit the interaction of the OIF P particle with multivalent Le^a^-tri-PAA conjugates, most likely due to avidity effects. Therefore, further investigations will be necessary to develop polymers that contain multivalent structures of glycerol molecules as antiviral candidates. Most importantly, our study should provide a solid structural basis and a model for future studies.

In summary, we have identified and proved the genetic branch of GII.13/21 as new evolutionary lineage with a novel binding interface for HBGAs. We have elucidated the structural basis for the abolishment of the conventional GII HBGA binding interface and the development of the new binding site. We also showed that the new HBGA binding interface is conserved among members of the GII.13/21 lineage, indicating that a new selection pressure has been asserted through the interaction with HBGAs. Finally, we have identified glycerol as a potential low-cost compound to become an antiviral candidate for future development. In conclusion, our data provide new insights into the complex interactions between the diverse huNoV and the polymorphic HBGAs.

## Materials and Methods

### Protein expression and purification

OIF (Operation Iraqi Freedom) virus is a GII.21 huNoV that was isolated from a huNoV outbreak in a US military deployed to Iraq in 2003. The cDNA sequences encoding the P domain of the OIF virus (accession number: AY675554; residues 220 to 527) was cloned into pGEX-6P-1 expression vector between the EcoR I and Xho I sites of the MCS. The OIF P domain was expressed as a GST fusion protein in *Escherichia Coli* BL 21 (DE 3) upon induction with 0.5 mM Isopropyl β-D-1-Thiogalactopyranoside (IPTG) at 16°C for 18 hours, at an OD_600 nm_ of 0.6–0.8. The recombinant protein was purified using glutathione-sepharose 4B (GE Healthcare Life Sciences) following the manufacturer’s guidelines. The GST-P domain fusion protein was cleaved on the resin by Prescission Protease (GE Healthcare Life Sciences) at 4 °C overnight, and the P proteins were further purified by Resource Q anion ion exchange (GE), using a buffer containing 20 mM HEPES (pH 7.5), with P domain protein eluted at approx. 100 mM NaCl.

### Crystallization of native P domain protein and its complex with Le^a^ trisaccharide

The purified native P domain protein was dialyzed against buffer containing 20 mM HEPES (pH 7.5), 150mM NaCl and 5% (v/v) glycerol, before it was concentrated to 13 mg/mL for crystallization. Native P domain crystals were grown by hanging-drop vapor diffusion method, with the crystallization droplet containing 1 μL protein and 1 μL reservoir solution containing 0.1 M MES (pH 6.5), 0.25 M (NH_4_)_2_SO_4_, 18% (w/v) polyethylene glycol (PEG) 3350. The crystals were grown at 16°C and harvested after approx. one week.

While performing the crystallization experiments, we observed that the presence of glycerol inhibited the co-crystallization of the OIF P domain-Le^a^ complex. The P protein used for co-crystallization with Le^a^-trisaccharide was purified with identical solutions (see above), but in the absence of glycerol. The Le^a^ trisaccharide [β-Gal-(1,3)-(α-Fuc-(1,4))- GlcNAc] (J&K, China) was dissolved in double distilled water and prepared as 20 mM solution, and then mixed with an equal volume of native P domain protein (26 mg/mL), and incubated at 4°C for 1 hour before crystallization. The final reservoir for the growth of complex crystals contained 0.25 M (NH_4_)_2_SO4, 18% (w/v) PEG 3350. Micro-seeding technique was used to aid the growth of complex crystal with native crystal seeds 16 hours after the setting of crystallization droplets. OIF P domain-Le^a^ complex crystals were harvested in three days.

### Data collection and processing, structure determination and refinement

The cryo-protectants of crystals of unliganded P domain and P domain-Le^a^ complex were the corresponding reservoir solutions complemented with 15% (v/v) PEG 400. Crystals were briefly soaked in the cryo-protectant for 5 seconds before being mounted for diffraction test. The diffraction data for native crystal were collected at the beamline 41U of SPRING8 (Japan) at a wavelength of 1.0000 Å, while the Le^a^ complex data were collected at rotating-anode X-ray source MicroMax-007/Satun 944 HG/Varimax HF (Institute of Biophysics, CAS, Beijing) at the wavelength of 1.5418 Å. Diffraction data were processed, scaled, and merged using the HKL-2000 program package [43]. Data collection statistics are summarized in Table 1.

**Table 1:** Statistics of data-collection.

Parameters	Native Protein	Complex with Le^a^-tri
Space group	P2_1_	P2_1_2_1_2_1_
Wavelength (Å)	1.0000	1.5418
Resolution (Å)^**a**^	50.00–1.19 (1.21–1.19)	50.00–2.00 (2.07–2.00)
Cell dimensions (Å)		
a, b, c (Å)	49.5, 112.7, 59.7	54.9, 84.2, 124.7
α, β, γ (°)	90, 108.5, 90	90, 90, 90
No. of Measured reflections	645932	216182
No. of Unique reflections	187824	37781
Completeness (%)^**a**^	94.9 (86.9)	94.7 (68.4)
Redundancy ^**a**^	3.4 (3.3)	5.7(2.6)
I/σ (I) ^**a**^	25.2 (4.1)	17.3 (3.7)
R _merge_ ^**a**^ ^,^ ^b^	0.056 (0.316)	0.073 (0.236)

a. Values in parentheses correspond to the shell of highest resolution.

b. R_merge_ = Σi|Ii–<I>|/ΣiIi, where Ii and <I> are the observed and mean intensity of related reflections with common indices h,k, and l.

The native crystal structures were solved by molecular replacement method using Phaser of CCP4 suite [44] and the GII.9 NoV VA207 P domain (PDB ID:3PUN) structure as the initial search model. Automatic structure building and refinement were carried out using Phenix program [45] and manual adjustment was done using the program COOT [46] with guidance of (2Fo-Fc) and (Fo-Fc) electron density maps. Water molecules were added at the final round of structure optimization at (Fo-Fc) electron density map peaks (>2.5 σ) where they can form stable hydrogen bonding with nearby amino acid residues. The phases and structures of the P domain-Le^a^ complex were solved using the final refined structure of native P domain protein as model. Structure refinement statistics are summarized in Table 2. The final structure validation was done with the PROCHECK [47], with no residue found at disallowed region of the Ramachandran plot. Structural analysis was performed using EdPDB [48] and Pymol [49].

**Table 2:** Statistics of structure refinement.

Parameters	Native Protein	Complex with Le^a^-tri
**No. of reflections in working set**	178343	35812
**No. of reflections in test set**	9430	1884
R_work_ ^**a**^	0.140	0.171
R_free_ ^b^	0.160	0.209
**Root mean square deviation (RMSD)**		
Bond lengths (Å)	0.005	0.007
Bond angles(°)	1.131	1.097
**Average B factors(Å** ^**2**^)		
Total	13.8	21.2
Protein	11.6	20.5
Ligand	15.2 (glycerol)	25.3
Solvent	25.0	26.7
**Ramachandran plot (%)**		
Favored	97.7%	95.9%
Allowed	2.3%	4.1%
Disallowed	0.0%	0.0%
**PDB ID**	4RLZ	4RM0

a. R_work_ = Σ||F_obs_|-|F_cal_|||/Σ|Fobs|, R_free_ = ΣT||F_obs_|-|F_cal_|||/ΣT|F_ob_s|, where F_obs_ and F_cal_ are observed and calculated structure factors, respectively.

b. R_free_, T is a randomly selected test data set (5%) of total reflections and was set aside before structure refinement.

### Production of OIF P particles containing single mutations

Single residue mutations were introduced to the HBGA binding site of the OIF P domain by site-directed mutagenesis using the expression plasmid of the wild type OIF P domain as template. Mutagenesis was carried out using the QuickChange Site-Directed Mutagenesis Kit (Agilent Technology, CA) and corresponding primer pairs containing the mutation sites. After confirmation of mutations, the P proteins were expressed as P particles and purified using an *E*. *coli* system (BL21) as described elsewhere [31, 50, 51]. The GST-P domain fusion proteins were cleaved by thrombin (GE Healthcare Life Sciences) to allow the P proteins to self-assemble into P particles. P particle formation was monitored by gel filtration through a size-exclusion column (Superdex 200, GE Healthcare Life Sciences) powered by an AKTA-FPLC system (model 920, GE Healthcare Life Sciences, Piscataway, NJ) followed by SDS-PAGE electrophoresis. The P particles showed a molecular weight of 830 kDa. None of the mutations in this study affected the formation of P particles, as confirmed by FPLC (data not shown). All mutant P particles can be well detected by the hyperimmune serum against norovirus VLPs that was used in the HBGA binding and blocking assays (below).

### HBGA binding and blocking assays

HBGA assays were carried out as described previously [5]. Briefly, a panel of synthetic oligosaccharides (GlycoTech, Gaithersburg, MD) representing types A, B, H1, H2, H3, Le^a^, Le^b^, Le^x^, Le^y^, sialyl Le^a^, and sialyl Le^x^ as well as the disaccharides representing the type 1 and type 2 precursors of HBGAs at 2 μg/mL were coated on microtiter plates at 4°C overnight. After blocking with 5% (w/v) non-fat milk, the coated oligosaccharides were incubated with the affinity-column purified OIF P particles (10 ng/μL) for 60 minutes at 37°C. The bound OIF P particles were detected as described previously, using an in-house hyperimmune rabbit serum against various huNoVs [5, 30]. For glycerol blocking assays, the OIF P particles (4 μg/mL) were mixed with the monomeric glycerol (1–10%) and incubated with the coated polyvalent Le^a^-tri-PAA (polyacrylamide) conjugates (2 μg/mL). The bound OIF P particles were detected as described above.

### Accession numbers

The cDNA sequences encoding the P domain of the OIF virus has been submitted to GenBank previously with an accession number of AY675554. Coordinates and structure factors of the native OIF P protein and the complex with Le^a^ trisaccharide have been deposited in the Protein Data Bank (www.pdb.org) with the pdb accession codes of 4RLZ and 4RM0, respectively. The GenBank accession numbers of the sequences that were used in Figs 1 and 8 were shown in their figure legends.
