# Correction: A Unique Human Norovirus Lineage with a Distinct HBGA Binding Interface

**DOI:** 10.1371/journal.ppat.1005599

**Published:** 2016-04-19

**Authors:** Wu Liu, Yutao Chen, Xi Jiang, Ming Xia, Yang Yang, Ming Tan, Xuemei Li, Zihe Rao

The following information is missing from the Funding section: This study was supported by the External Cooperation Program of the Chinese Academy of Sciences (Grant No. GJHZ1405).
